# The Impact of Treatment Delay on Endometrial and Ovarian Cancer Patients: A Systematic Review

**DOI:** 10.3390/cancers17132076

**Published:** 2025-06-21

**Authors:** Dimitrios Zouzoulas, Tilemachos Karalis, Iliana Sofianou, Christos Anthoulakis, Katerina Tzika, Menelaos Zafrakas, Eleni Timotheadou, Grigoris Grimbizis, Dimitrios Tsolakidis

**Affiliations:** 1 1^st^ Department of Obstetrics & Gynecology, Aristotle University of Thessaloniki, “Papageorgiou” Hospital, 56403 Thessaloniki, Greece; 2 Department of Oncology, Aristotle University of Thessaloniki, “Papageorgiou” Hospital, 56403 Thessaloniki, Greece

**Keywords:** ovarian cancer, endometrial cancer, treatment delay, survival outcomes, quality of life

## Abstract

Background/Objectives: Ovarian and endometrial cancers present significant health burdens due to their substantial morbidity and mortality. Surgery delay is hypothesized to negatively impact patient outcomes, yet evidence remains inconsistent. This systematic review aims to comprehensively evaluate current evidence regarding the effects of treatment delay on survival and quality of life in these patients. Methods: A systematic literature search was conducted in MEDLINE (PubMed), Cochrane Library, Scopus, and ClinicalTrials.gov databases, complemented by a manual screening of reference lists, with studies included up to March 2025. Two independent reviewers screened and assessed the methodological quality using the ROBINS-E tool and extracted the data from the studies. Due to substantial methodological and clinical heterogeneity, results were synthesized qualitatively through narrative synthesis. Results: Twenty-one studies met the inclusion criteria, comprising predominantly retrospective cohort analyses. Treatment delays consistently correlated with decreased survival and advanced disease stages. Among studies, optimal intervals from diagnosis to treatment were varied from 2 to 8 weeks. Patient-reported outcomes indicated heightened anxiety, increased distress, and poor satisfaction with prolonged delays. Healthcare system factors such as resource allocation, geographic disparities, and socioeconomic inequalities significantly contributed to delays. Quantitative survival metrics supported a clear negative association between treatment delays and survival, although paradoxical outcomes in select subgroups highlighted underlying complexities. Conclusions: This systematic review underscores the critical importance of minimizing delays in the treatment of ovarian and endometrial cancers to improve patient survival and psychological outcomes. Identified healthcare system barriers demand targeted policy interventions to ensure timely and equitable access to tertiary healthcare infrastructures. Future research should prioritize standardizing delay definitions to strengthen and homogenize evidence.

## Simple Summary

Timely treatment is crucial for improving survival in endometrial and ovarian cancer patients. This systematic review aims to comprehensively evaluate current evidence regarding the effects of treatment delay on survival and quality of life in these patients. Data from twenty-one studies were analyzed to better understand how delays from diagnosis to treatment affect the survival and quality of life of these patients. We found that longer delays generally worsen survival rates, lead to a progression in stage, and lead to a deterioration in patients’ quality of life. Healthcare system factors, such as financial resources and geographic location, significantly influence these delays. Although differences in study designs and results exist, clearer guidelines and standardized approaches are necessary for future research.

## 1. Introduction

### 1.1. Rationale

Ovarian and endometrial cancers represent two of the most significant gynecologic malignancies, posing considerable health challenges globally due to their high morbidity and mortality rates [1]. Ovarian cancer is a silent women killer, because it typically presents with nonspecific symptoms often leading to advanced-stage diagnosis, which significantly reduces treatment effectiveness and survival prospects [2]. On the other hand, endometrial cancer tends to present with more noticeable clinical symptoms, such as abnormal uterine bleeding, which often facilitates earlier detection and potentially better prognosis [3]. Despite this, substantial delays between the onset of initial symptoms or formal diagnosis and initiation of definitive treatment persist in both conditions [4].

Treatment delay, defined as the time interval from initial symptom presentation or histological confirmation of disease to the initiation of therapeutic intervention, has been extensively studied; however, findings remain inconsistent. Clinically, it is often presumed that prolonged delays may result in tumor progression or metastasis, thus negatively influencing patient outcomes in various cancer types, including survival rates and quality of life [4]. For instance, the systematic review by Neal et al. regarding breast cancer suggests delays of 3–6 months were associated with lower survival, whereas for bladder cancer evidence suggests a potential negative impact on outcomes associated with delays [4]. However, there are reports with contradictory results, with some studies suggesting detrimental effects of prolonged waiting lists, while other studies demonstrate no clear adverse outcomes related to extended delays [5]. This variability indicates the influence of additional factors such as inherent tumor biology, disease stage at diagnosis, and healthcare system characteristics [6].

Given the complexity and inconsistency observed in the existing literature there is a critical need to systematically examine evidence specifically addressing the impact of delays in initiating treatment for ovarian and endometrial cancers. Prior reviews have broadly addressed delays across multiple cancer types, highlighting the necessity for a focused systematic review targeting these two gynecologic malignancies to better understand the clinical implications of treatment delays.

### 1.2. Objectives

The primary objective of this systematic review is to comprehensively evaluate the existing evidence regarding the effects of delay from the initial symptom onset or formal diagnosis to the initiation of treatment in ovarian and endometrial cancer, especially regarding survival outcomes and quality of life. Additionally, this review aims to identify and discuss potential reasons behind inconsistencies observed across different studies, including variations in tumor biology, patient demographics, and differences within healthcare delivery systems. Ultimately, this systematic review intends to provide clear, evidence-based recommendations that can guide clinical decision-making, optimize treatment timelines, and improve outcomes for patients diagnosed with ovarian and endometrial cancers.

## 2. Materials and Methods

### 2.1. Eligibility Criteria

Eligible studies for inclusion in this systematic review were observational studies (cohort studies, retrospective studies, and case–control studies) specifically investigating delays from initial symptom onset, histological diagnosis, or formal clinical diagnosis to the initiation of treatment (surgical intervention, chemotherapy, or radiotherapy) in patients diagnosed with ovarian or endometrial cancer. Included studies were required to report clear outcome data regarding survival outcomes (overall survival and disease-specific survival), disease progression, the stage of disease at diagnosis, patient quality of life, or patient satisfaction with healthcare received. Studies were excluded if they exclusively reported on other gynecological cancers or malignancies without separate data for ovarian or endometrial cancers. Case reports or case series with fewer than 10 patients, editorials, reviews, conference abstracts, or studies without clear documentation of delay intervals or relevant clinical outcomes were also excluded. For studies conducted at the same institution with overlapping data, only the most recent or methodologically robust study was included to prevent data duplication.

### 2.2. Information Sources

A thorough literature search was conducted to identify relevant studies published in peer-reviewed journals. Databases searched included MEDLINE via PubMed, Cochrane Library, Scopus, and Clinicaltrials.gov registries. Additionally, reference lists from identified articles and relevant systematic reviews were screened manually to locate further eligible studies. No date restrictions were applied, and only articles published in English were included. Grey literature, including conference proceedings, dissertations, and unpublished studies, was excluded from this review.

### 2.3. Search Strategy

Our search strategy is presented below:

- PubMed (999 studies):

(“Endometrial Neoplasm” OR “Endometrial Carcinoma” OR “Endometrium Cancers” OR “Endometrial Neoplasms”[Mesh] OR “Ovarian Neoplasm” OR “Ovarian Cancer” OR “Ovarian Carcinoma” OR "Ovarian Neoplasms”[Mesh]) AND (“Time-to-Treatment” OR “Treatment Delay” OR “Delay” OR “Time-to-Treatment”[Mesh]).

- Cochrane (386 studies):

((“Endometrial Neoplasm” OR “Endometrial Carcinoma” OR “Endometrium Cancers” OR “Ovarian Neoplasm” OR “Ovarian Cancer” OR “Ovarian Carcinoma”) AND (“Time-to-Treatment” OR “Treatment Delay” OR “Delay”)) in Title Abstract Keyword.

- Scopus (1767 studies):

TITLE-ABS-KEY ((“Endometrial Neoplasm” OR “Endometrial Carcinoma” OR “Endometrium Cancers” OR “Ovarian Neoplasm” OR “Ovarian Cancer” OR “Ovarian Carcinoma”) AND (“Time-to-Treatment” OR “Treatment Delay” OR “Delay”)).

- ClinicalTrials.org (134 studies):

Condition: Gynecologic Cancer/Other Terms: Time to surgery.

Search results were updated up to the 1st of March 2025. The filters that were applied were English language and human female population.

### 2.4. Selection Process

Two independent reviewers initially screened the title and abstract of the retrieved studies to identify articles potentially eligible for inclusion. Following this, full-text evaluations of these selected studies were independently conducted by both reviewers to determine final eligibility. Discrepancies during the selection process between the reviewers were resolved through discussion and consensus involving a third investigator.

### 2.5. Data Collection Process

Data from eligible studies were independently extracted by two authors using a standardized form designed specifically for this systematic review. Discrepancies between authors during data extraction were resolved through discussion and consensus; in cases where consensus could not be reached, a third author was consulted to facilitate a resolution. When additional clarification was required, attempts were made to contact the corresponding authors of the original studies via email. Extracted data were systematically recorded into pre-formulated tables to ensure completeness and accuracy, facilitating subsequent synthesis and analysis.

### 2.6. Data Items

The following data items were extracted from each included study: authors, the year of publication, study design, geographical location, study setting (hospital-based or population-based), the number of participants, participant demographics (age, ethnicity, and socioeconomic status), cancer type (ovarian or endometrial), cancer stage and histological subtype, the definition and measurement of delay intervals (from symptom onset or diagnosis to treatment initiation), the type of treatment received (surgery, chemotherapy, or radiotherapy), outcomes assessed (overall survival, disease-specific survival, disease progression, patient quality of life, and patient satisfaction), and any reported statistical measures (hazard ratios, odds ratios, survival rates, or other relevant measures).

### 2.7. Study Risk of Bias Assessment

Two independent reviewers assessed the risk of bias for each included study using the Risk Of Bias In Non-randomized Studies of Exposures (ROBINS-E) tool [7]. The ROBINS-E tool evaluates studies based on domains including bias due to confounding, participant selection, the classification of exposures, deviations from intended exposures, missing data, the measurement of outcomes, and the selection of the reported result. Each domain was evaluated and rated as having a low, moderate, serious, or critical risk of bias. Overall bias judgments were determined by the highest risk observed in any domain. Disagreements between reviewers were resolved through discussion, and when necessary, by consultation with a third reviewer. Results of the risk of bias assessment were transparently reported to inform the interpretation of the review findings.

### 2.8. Effect Measures

This systematic review focused on the following effect measures: hazard ratios (HRs) for survival outcomes (overall and disease-specific survival), odds ratios (ORs) for disease progression or stage advancement from diagnosis, and mean or median differences for patient-reported outcomes, including quality of life and patient satisfaction. Effect measures were selected based on their availability and consistency across the included studies, facilitating both quantitative data extraction and narrative synthesis of the review findings. Whenever available, 95% confidence intervals (CIs) and *p*-values were extracted alongside the primary effect measures to assess the strength and statistical significance of the observed associations.

### 2.9. Synthesis Methods

A narrative synthesis approach was employed to summarize and analyze findings from the included studies. Due to the anticipated clinical and methodological heterogeneity among studies, including variations in definitions of delay intervals, patient characteristics, outcome measures, and study designs, a quantitative synthesis (meta-analysis) was not initially conducted. Instead, results were synthesized qualitatively, grouped by cancer type (ovarian or endometrial), delay intervals (symptom onset or diagnosis to treatment), and outcomes assessed. Key findings and patterns, including consistencies and inconsistencies across studies, were highlighted. If a sufficient number of methodologically homogeneous studies reporting comparable outcomes were identified during the synthesis, a meta-analysis would be considered to quantitatively combine results using a random-effects model. This potential meta-analysis would involve a statistical heterogeneity assessment using I^2^ statistics and the visual inspection of forest plots.

### 2.10. Reporting Bias Assessment

Reporting bias was assessed qualitatively due to the expected heterogeneity in study designs and outcomes reported. Two reviewers independently evaluated the potential for selective outcome reporting by comparing study protocols (when available) or methods sections with the reported results. If discrepancies or a selective reporting of outcomes were suspected, attempts were made to clarify these through direct contact with study authors. Funnel plots and statistical tests for reporting bias were planned for use if a meta-analysis was conducted and a sufficient number of studies (>10) with comparable outcomes were available. The results of the qualitative reporting bias assessment were transparently described to provide context for interpreting the review findings.

### 2.11. Certainty Assessment

If the results of the studies allowed it, the certainty of evidence for each outcome assessed would be evaluated using the Grading of Recommendations Assessment, Development, and Evaluation (GRADE) approach [8]. The GRADE method systematically assesses factors such as risk of bias, inconsistency, indirectness, imprecision, and publication bias, categorizing the quality of evidence into four levels: high, moderate, low, and very low. Two reviewers would independently perform the GRADE assessments and any disagreements would be resolved through discussion or consultation with a third reviewer.

## 3. Results

### 3.1. Study Selection

The comprehensive literature search resulted in a total of 3286 records across four databases: PubMed (999 studies), Cochrane Library (386 studies), Scopus (1767 studies), and ClinicalTrials.org (134 studies). Following the initial removal of duplicate entries (431 studies) and exclusions due to predefined criteria (language other than English, non-human studies, or non-female populations; 196 studies), 2659 studies remained for screening based on title and abstract.

**Figure 1:**
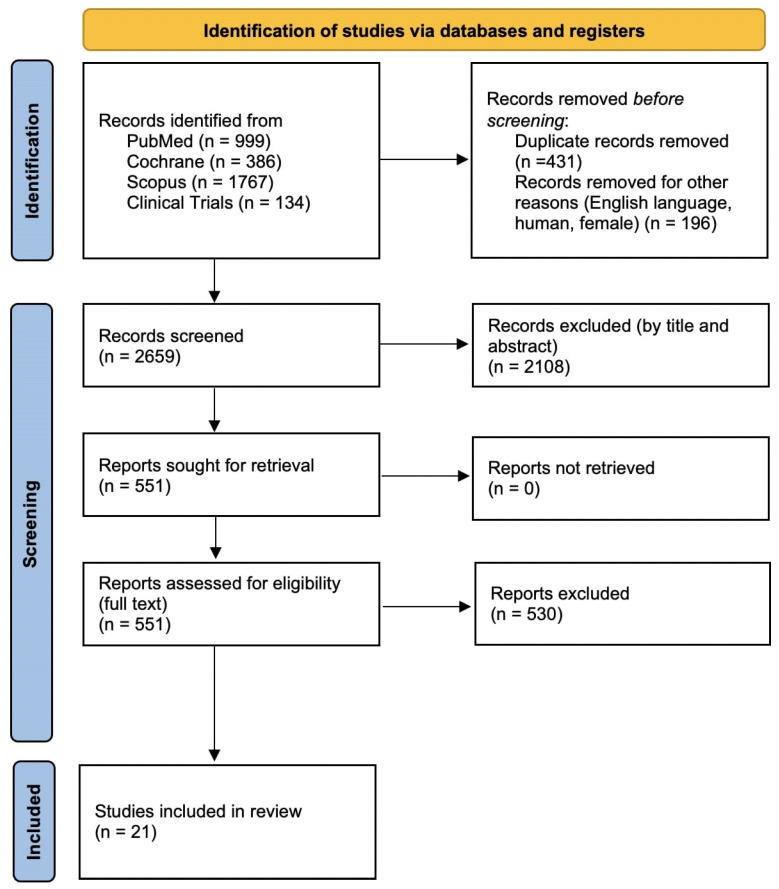
PRISMA flowchart.

From these 2659 records, a further 2108 were excluded upon preliminary screening, as they clearly did not meet the eligibility criteria. Consequently, 551 records underwent a detailed, full-text assessment to evaluate their relevance and eligibility based on the predefined inclusion and exclusion criteria. This full-text screening resulted in the exclusion of an additional 530 studies. Ultimately, 21 studies were selected for inclusion in this systematic review (AlHilli et al. 2019 [9], Crawford et al. 2002 [10], Elit et al. 2014 [11], Frey et al. 2016 [12], Frey et al. 2020 [13], Huepenbecker et al. 2022 [14], Kadan et al. 2020 [15], Levin et al. 2024 [16], Marcickiewicz et al. 2022 [17], Matsuo et al. 2015 [18], Mitric et al. 2020 [19], Nica et al. 2022 [20], O’Leary et al. 2013 [21], Rabiu et al. 2013 [22], Robinson et al. 2012 [23], Sabourin et al. 2015 [24], Shalowitz et al. 2017 [25], Strohl et al. 2016 [26], Hansen et al. 2011 [27], Zouzoulas et al. 2024 [28], Zouzoulas et al. 2025 [29]). This systematic review follows the PRISMA guidelines [30] and the detailed flowchart summarizing this selection process is presented in Figure 1.

### 3.2. Study Characteristics

The twenty-one studies included in this systematic review consisted primarily of retrospective observational cohort designs, with a combination of population-based and hospital-based settings. Studies originated from diverse geographic regions, including the United States, Canada, Australia, United Kingdom, Sweden, Denmark, Germany, Greece, Israel, and Nigeria, thus reflecting varied healthcare systems and practices.

Overall, sixteen studies focused specifically on endometrial cancer, while seven addressed ovarian cancer, with two studies (Robinson et al. [23]; Frey et al. [12]) examining both cancers in distinct subgroups. Sample sizes varied substantially across studies, ranging from relatively small cohorts of 37 patients (Rabiu et al. [22]) to large national database analyses including over 200,000 patients (Shalowitz et al. [25]). Delay intervals assessed varied among studies, including delays from symptom onset to treatment, diagnosis to treatment, or both. Treatment modalities evaluated primarily encompassed surgical interventions, chemotherapy, and radiotherapy, either alone or in combination. Detailed characteristics of the included studies are summarized separately in Table 1 (endometrial cancer studies) and Table 2 (ovarian cancer studies).

### 3.3. Risk of Bias in Studies

The risk of bias across the twenty-one included studies was systematically assessed using the ROBINS-E tool [7], evaluating each study across multiple bias domains including bias due to confounding, exposure measurement, participant selection, post-exposure interventions, missing data, outcome measurement, and the selection of reported results.

Most studies (18 out of 21) were judged to have a moderate overall risk of bias, indicating some concerns, but without severely undermining confidence in the findings. The primary reasons for moderate bias ratings typically included potential confounding and moderate bias related to participant selection and exposure measurement. A smaller subset of studies (three studies) was classified as having a serious overall risk of bias, primarily due to significant confounding, serious issues in participant selection, and biases in exposure or outcome measurement. These studies, Crawford et al. [10], Hansen et al. [27], and Rabiu et al. [22], were identified to have serious overall bias due to methodological limitations and an inadequate control of confounding factors, while the majority of large-scale, population-based studies demonstrated a more robust methodological rigor and lower bias ratings.

**Figure 2:**
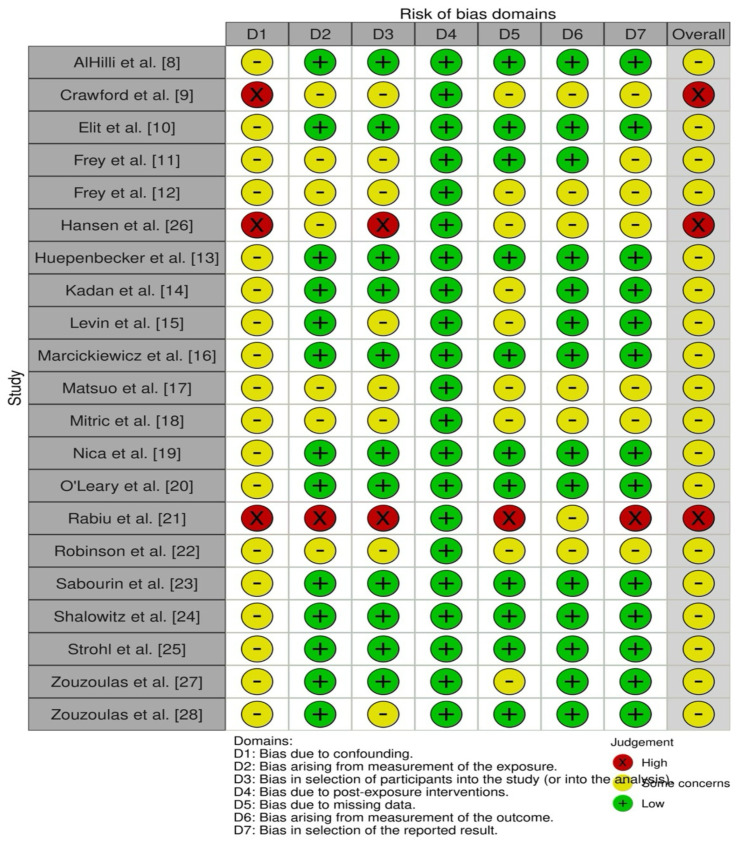
Traffic light plot of risk of bias [8,9,10,11,12,13,14,15,16,17,18,19,20,21,22,23,24,25,26,27,28].

**Figure 3:**
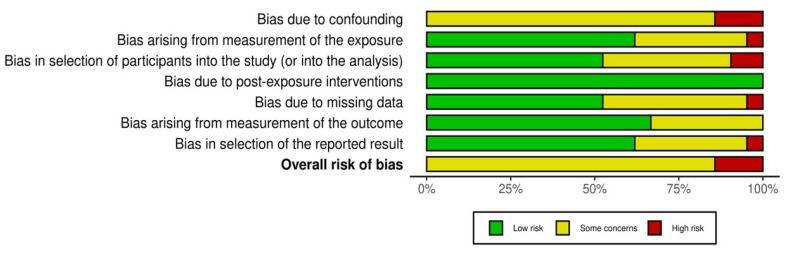
Summary plot of risk of bias.

A comprehensive summary of the risk of bias assessments for each individual study across all domains is provided in the following figures (Figure 2 and Figure 3).

### 3.4. Results of Individual Studies

The results of the studies are presented below, in Table 3.

#### 3.4.1. Quantitative Data

Among the included studies, eleven provided clearly reported quantitative data on survival outcomes associated with delays in treatment for endometrial or ovarian cancer patients, although each in their own way and with heterogenous methods and outcome measures. These studies were AlHilli et al. [9], Shalowitz et al. [25], Strohl et al. [26], Crawford et al. [10], Elit et al. [11], Matsuo et al. [18], Mitric et al. [19], Nica et al. [20], Sabourin et al. [24], Zouzoulas et al. [28], and Zouzoulas et al. [29]. Their results are summarized in the following table (Table 4).

Quantitative results from these studies consistently supported the association between increased delays and worse survival outcomes in patients with endometrial cancer. AlHilli et al. [9] found a significant hazard ratio (HR) of 1.22 for increased mortality in stage I-II endometrial cancer patients experiencing delays exceeding six weeks. Interestingly, they reported paradoxically improved survival outcomes for patients with stage IV disease and prolonged treatment delays, indicating complexities possibly related to patient selection or disease severity. Shalowitz et al. [25] indicated elevated mortality risks for surgeries conducted too soon (<2 weeks) or after significant delays (>8 weeks), pointing to the necessity of optimal timing. Similarly, Strohl et al. [26] found a clear overall survival impairment with treatment delays beyond six weeks (HR 1.14). Elit et al. [11] demonstrated significantly poorer 5-year survival outcomes for treatment intervals exceeding 12 weeks or less than two weeks, suggesting the need for balanced timing. Interestingly, Crawford et al. [10] paradoxically found reduced hazard ratios for longer delays (>62 days), likely due to selection biases or patient characteristics influencing treatment urgency. 

Furthermore, Matsuo et al. [18] and Mitric et al. [19] reported no significant survival differences across various delay intervals, underscoring the complexity of evaluating the impact of delays and the potential role of tumor biology and patient demographics. Specifically, low-risk endometrial cancers may be less adversely affected by moderate treatment delays compared to high-risk cancers which are more likely to result in worse outcomes. Nica et al. [20] identified progressively worse survival outcomes with longer intervals from oncology appointments to surgery (>45 days), reinforcing the negative impacts of prolonged delays. Sabourin et al. [24] found significantly decreased 5-year overall survival for delays exceeding 12 weeks. Similarly, Zouzoulas et al. [28] reported worse disease-free survival with delays exceeding 8 weeks from initial diagnosis, whereas the overall survival was not significantly impacted. Finally, in the relatively small study of Zouzoulas et al. [29], the only study with quantitative data on ovarian cancer that reported DFS and OS, their respective values showed no significant impact. Overall, these studies provide evidence pointing to the causative relationship between treatment delays and patient survival, despite certain paradoxical findings that underline the complexities inherent to clinical practice and patient management.

Although there is a group of studies included in this review that provided quantitative data regarding survival outcomes, it was decided not to perform a meta-analysis due to the considerable methodological and clinical diversity among them. Variations in definitions and measurements of treatment delays, ranging from symptom onset or clinical suspicion to formal diagnosis, created substantial heterogeneity. Additionally, studies differed markedly in reported outcomes, with inconsistencies in survival metrics, such as overall survival, disease-specific survival, and progression-free intervals, and often lacking uniformly reported hazard ratios or confidence intervals. Patient populations also varied significantly regarding disease stage, cancer type, healthcare system contexts, and demographic characteristics. Due to these factors, the statistical pooling of results was methodologically inappropriate and could potentially produce misleading interpretations. Therefore, a qualitative narrative synthesis was conducted, providing clarity and preserving the integrity of the conclusions drawn from the available data.

#### 3.4.2. Delay Intervals and Measurement Methods

The studies included in this review demonstrated considerable variability regarding the definition and measurement of treatment delays. Most studies (AlHilli et al. [9]; Elit et al. [11]; Marcickiewicz et al. [17]; Shalowitz et al. [25]; Strohl et al. [26]; Zouzoulas et al. [28]) defined delay intervals as the time from histologic or clinical diagnosis to the initiation of treatment, typically surgery. Others specifically focused on intervals beginning from biopsy-confirmed diagnoses (Kadan et al. [15]; Levin et al. [16]; Matsuo et al. [18]; Mitric et al. [19]). A smaller subset of studies (Hansen et al. [27]; Rabiu et al. [22]; Robinson et al. [23]; Huepenbecker et al. [14]; Zouzoulas et al. [29]) measured delays from the onset of the initial symptoms reported by patients to treatment initiation, thereby capturing patient-related delays and healthcare system responsiveness.

Differences were also noted in the specificity of the reported delays: some studies provided broad intervals or median days, while others precisely defined cutoff points (e.g., >6 weeks, >8 weeks, or >12 weeks), explicitly relating them to survival or clinical outcomes. Additionally, Frey et al. [13] uniquely assessed treatment delays due to disruptions caused by the COVID-19 pandemic, focusing specifically on delays in scheduled treatment rather than delays from diagnosis or symptom onset. This methodological heterogeneity across studies necessitated a careful narrative synthesis to accurately interpret and contextualize their results.

#### 3.4.3. Impact of Delay in Survival Outcomes

Numerous studies evaluated the relationship between treatment delays and survival outcomes. Several large-scale studies, including AlHilli et al. [9], Elit et al. (2014), Shalowitz et al. [25], and Strohl et al. [26], consistently reported that prolonged delays from diagnosis to treatment initiation negatively impacted overall survival. Delays exceeding certain thresholds, often cited as six to eight weeks, were frequently associated with significantly lower survival rates. Zouzoulas et al. [28] further supported this finding by reporting significantly worse disease-free survival for endometrial cancer patients experiencing delays longer than eight weeks, though overall survival differences were not statistically significant, while Shalowitz et al. [25] identified an optimal interval of three to eight weeks from diagnosis to surgery, highlighting both shorter (<2 weeks) and longer (>8 weeks) intervals as detrimental to survival.

Conversely, a smaller subset of studies, Matsuo et al. [18] and Mitric et al. [19], reported no significant survival disadvantage linked to delays, suggesting a more complex relationship possibly moderated by tumor biology or patient characteristics. Similarly, Marcickiewicz et al. [17] reported paradoxical results, indicating that extremely short intervals (<2 weeks) from diagnosis to surgery were also associated with poorer survival outcomes, raising questions about potential confounders (patient selection bias) and disease severity influencing these findings. Potential explanations for this phenomenon may include inadequate preoperative medical optimization, an insufficient evaluation of patient comorbidities, clinical urgency due to advanced disease presentation, and possibly, treatment being conducted in lower-volume facilities with fewer resources. Finally, Zouzoulas et al. [29], examining early-stage ovarian cancer patients, reported no significant differences in survival with delays greater than five weeks, further complicating the narrative regarding delay intervals and survival outcomes. The mixed evidence underscores the necessity of considering clinical context and patient-specific factors when interpreting the relationship between treatment delay and survival.

#### 3.4.4. Impact of Delay on Disease Progression and Cancer Stage

Several included studies specifically explored the relationship between delays in treatment initiation and disease progression or cancer staging at the time of treatment. Robinson et al. [23] and Rabiu et al. [22] highlighted significant associations between prolonged delays from initial symptom onset and more advanced disease stage at treatment, emphasizing the detrimental role of extended delays in facilitating disease progression. Zouzoulas et al. [28] identified a significantly increased requirement for adjuvant pelvic radiation in patients whose surgical treatments were delayed more than eight weeks, reflecting more adverse pathological features due to prolonged delays. Likewise, Hansen et al. [27] demonstrated that prolonged intervals from symptom onset to definitive treatment significantly correlated with more advanced cancer stages, suggesting that timely diagnosis and management are critical to mitigate disease advancement.

On the other hand, other studies (Kadan et al. [15]; Levin et al. [16]; and Zouzoulas et al. [29]) reported minimal or no significant association between treatment delays and stage progression, particularly in lower-risk patient populations, thus suggesting a more nuanced impact of delay influenced by initial tumor biology or clinical presentation.

#### 3.4.5. Delay and Patient-Reported Outcomes

Only two studies specifically explored how treatment delays impacted patient-reported outcomes, particularly quality of life, anxiety, and overall patient satisfaction. Robinson et al. [23] and Frey et al. [13] highlighted significant negative effects of prolonged delays on patient quality of life and psychological well-being. Robinson and colleagues noted that patients experiencing extended delays reported higher levels of distress and reduced satisfaction with healthcare services, emphasizing the profound psychological and emotional burden imposed by uncertainty and prolonged waiting periods. Moreover, Frey and colleagues further reinforced these findings by investigating delays specifically attributed to the COVID-19 pandemic, documenting heightened anxiety and diminished quality of life in patients whose treatments were postponed. This study particularly underscored the significant psychological impact of delays during an unprecedented healthcare disruption.

Furthermore, patient satisfaction related to healthcare delivery appeared influenced by clear communication and active management of expectations rather than solely delay duration. This finding suggests that timely and transparent communication might alleviate some negative psychological impacts of treatment delays, emphasizing the importance of holistic patient care beyond shorter time intervals.

#### 3.4.6. Healthcare System Factors and Treatment Delays

There were a few studies in this review that explicitly explored how factors within healthcare systems contribute to treatment delays. Frey et al. [12] compared delays in treatment initiation between patients managed in public versus private healthcare facilities, highlighting significant differences, with notably longer intervals observed in the public sector. This discrepancy underscored potential resource constraints and differences in healthcare accessibility influencing treatment timeliness. Similarly, O’Leary et al. [21] and Nica et al. [20] examined regional disparities within healthcare delivery systems. Their findings indicated that patients residing in remote or rural regions frequently encountered longer delays from diagnosis to definitive surgical treatment compared to those in urban centers. Both studies emphasized that these delays might result from limited specialist availability and logistical challenges inherent to rural healthcare settings.

Shalowitz et al. [25] and Strohl et al. [26], using extensive national databases, further demonstrated demographic disparities, revealing that socioeconomic status and racial or ethnic backgrounds could significantly affect timely access to treatment. Patients from minority groups or lower socioeconomic backgrounds were disproportionately impacted by longer waiting periods, highlighting critical areas requiring targeted policy interventions and resource allocation to address systemic inequalities. Collectively, these studies underscore the complex interplay between healthcare infrastructure, resource allocation, and sociodemographic factors, illustrating how systemic elements within healthcare delivery significantly influence timely access to cancer treatments.

#### 3.4.7. COVID-19 Pandemic and Treatment Delay

The COVID-19 pandemic introduced unprecedented disruptions in healthcare services, significantly influencing treatment timelines for various medical conditions, including ovarian and endometrial cancers. Frey et al. [13] specifically assessed the impact of the pandemic-related delays, revealing substantial interruptions in scheduled cancer treatments. The study highlighted increased patient anxiety, uncertainty, and deteriorating quality of life resulting from postponed treatments.

These pandemic-related delays differed markedly from typical systemic delays, primarily driven by acute resource reallocation, the temporary closure of elective surgical services, and restrictions in outpatient and inpatient care. Such delays often lacked predictability and transparency, exacerbating patient distress and potentially affecting clinical outcomes. The findings underscore the necessity of robust healthcare contingency planning, clear patient communication, and efficient resource management to mitigate adverse effects during future healthcare crises.

#### 3.4.8. Comparisons Between Ovarian and Endometrial Cancer

Frey et al. [12] reported that while both ovarian and endometrial cancer patients experienced significant treatment delays, the implications varied between the two malignancies. Delays in ovarian cancer often corresponded to more pronounced impacts on patient survival outcomes, likely due to the biologically aggressive and rapidly progressing nature of ovarian tumors. Similarly, Robinson et al. [23] observed distinct variations, emphasizing that ovarian cancer patients generally presented with more advanced disease stages following prolonged symptom-to-treatment intervals compared to patients with endometrial cancer. This difference underscores the importance of timely intervention in ovarian cancer, where delays markedly increased the risk of disease progression and poorer prognosis.

Delays in endometrial cancer treatment were frequently associated with subtle impacts on survival and were more prominently connected to variations in patient-reported outcomes such as psychological distress and reduced satisfaction with healthcare delivery. Collectively, these comparative findings highlight critical differences in clinical implications and management strategies necessary for optimizing treatment timelines for each cancer type.

#### 3.4.9. Discrepancies and Conflicting Findings

This review identified several discrepancies and conflicting results among the included studies, reflecting complexities in evaluating the impact of treatment delays. For example, Marcickiewicz et al. [17] paradoxically found that both excessively short (<2 weeks) and prolonged intervals (>8 weeks) from diagnosis to surgery were associated with worse survival outcomes. This unexpected finding might reflect underlying clinical factors such as disease aggressiveness, patient comorbidities, or the urgency of surgical intervention, complicating the interpretation of delay intervals.

In addition, Matsuo et al. [18] and Mitric et al. [19] reported no clear survival disadvantage in longer delays, which contrasts sharply with numerous large-scale studies linking extended delays with poorer outcomes. Similarly, Zouzoulas et al. [29] observed no significant impact of delay intervals greater than five weeks on survival for early-stage ovarian cancer. Such conflicting evidence could be attributed to differences in patient selection, variations in tumor biology, or methodological discrepancies in study designs and outcome definitions.

These discrepancies highlight the importance of cautious interpretation and emphasize the need for context-sensitive analyses that consider patient-specific and healthcare system-related factors to accurately understand and mitigate the consequences of treatment delays.

#### 3.4.10. Reporting Biases

The potential for reporting biases was carefully assessed qualitatively due to the inherent heterogeneity of study designs and outcomes in the included studies. The majority of the studies transparently reported primary outcomes related to survival, disease progression, or delays in treatment. However, discrepancies were noted in the reporting of secondary outcomes, such as patient-reported measures of quality of life, psychological impact, and patient satisfaction. Several studies did not clearly specify whether all planned outcomes were fully reported, which limited the ability to fully assess potential selective reporting biases.

Funnel plot analyses or quantitative assessments of publication bias could not be performed due to the insufficient number of studies reporting comparable statistical outcomes. Nevertheless, careful examination of included studies did not reveal evident patterns indicative of systematic reporting bias. Despite these observations, the possibility of selective reporting, particularly in studies relying on registry-based data or large national databases, cannot be entirely ruled out. These limitations highlight the necessity for cautious interpretation of synthesized results and advocate for improved transparency in reporting future research.

#### 3.4.11. Certainty of Evidence

While the PRISMA guidelines recommend performing a formal certainty of evidence assessment using frameworks such as GRADE, the substantial methodological variability, heterogeneity in reported outcomes, and inconsistencies across studies included in this systematic review significantly limit the utility of such an assessment. The variability in measurement intervals, diverse outcome metrics, and substantial differences in patient populations and healthcare systems further reduce the applicability of a standardized certainty evaluation.

Consequently, the overall certainty of evidence derived from this review is inherently limited, with most evidence likely rated as low or very low certainty if formally assessed. Therefore, rather than providing a potentially misleading quantitative certainty rating, this review transparently acknowledges these inherent methodological limitations. Future research should aim for standardized methodologies and clearer reporting practices to enable more robust certainty assessments and provide stronger evidence to guide clinical and policy strategies for more beneficial healthcare systems.

## 4. Discussion

### 4.1. Summary of Main Findings

This systematic review synthesized evidence from twenty-one observational studies examining the effects of treatment delays in ovarian and endometrial cancers. The included studies consistently demonstrated significant variability in the delay time interval, ranging from initial symptom onset to diagnosis or from diagnosis to treatment initiation, highlighting methodological differences that complicated the interpretation of results. Nonetheless, several key themes emerged clearly across the studies.

One major finding was the frequent association between longer treatment delays and poorer survival outcomes, particularly evident in studies involving large national databases and robust patient cohorts. Delays extending beyond a critical threshold, typically cited between six to eight weeks from diagnosis to treatment initiation, were generally associated with decreased overall and disease-specific survival. Interestingly, certain studies also suggested negative outcomes associated with excessively short intervals, hinting at the complexity of optimal treatment timing and possibly reflecting underlying severity or the urgency of disease severity. Studies evaluating the relationship between delays and cancer staging consistently indicated that prolonged intervals contributed to disease progression, especially in ovarian cancer patients. The aggressive nature of ovarian malignancies appeared to exacerbate the detrimental effects of delayed intervention, leading to significantly worse prognoses for patients who experienced lengthy delays from initial symptom recognition to definitive treatment.

Another noteworthy finding from this review was the marked impact of delays on patient-reported outcomes. Delays were frequently linked to increased psychological distress, higher anxiety levels, and decreased overall patient satisfaction with healthcare services. This underscores the critical importance of timely communication and the proactive management of patient expectations as part of comprehensive cancer care.

Healthcare system-related factors emerged as pivotal influences on treatment timeliness. Variations between public and private hospital systems, regional disparities, and socioeconomic inequalities significantly contributed to observed delays. Patients in public healthcare settings, remote geographical locations, or lower socioeconomic groups frequently experienced notably longer waiting periods. This finding highlights social inequities that require targeted policy interventions and improved resource allocation strategies. Additionally, the unprecedented disruptions caused by the COVID-19 pandemic significantly impacted cancer treatment timelines. Patients faced extensive, often unpredictable delays, exacerbating anxiety and potentially worsening clinical outcomes. This highlighted the importance of robust contingency planning and transparent patient–provider communication during healthcare emergencies.

Lastly, discrepancies and conflicting findings across several studies added complexity to the overall interpretation. Some studies found minimal or no adverse effects from treatment delays, particularly for endometrial cancer or low-risk patient subsets. These inconsistencies emphasize the need to consider patient-specific factors, tumor biology, and methodological rigor when interpreting the impact of delays.

Overall, these summarized findings provide valuable insights into how treatment delays impact survival, disease progression, psychological well-being, and patient satisfaction, underscoring the critical need for timely and equitable cancer care interventions.

### 4.2. Clinical Implications of Treatment Delays

The findings from this systematic review carry significant implications for clinical practice, particularly regarding the timing and prioritization of treatment in ovarian and endometrial cancers. The clear association between prolonged delays and reduced survival outcomes highlights the critical necessity for clinicians to adhere to evidence-based optimal intervals from diagnosis to treatment initiation. For ovarian cancer, the clinical urgency is notably heightened due to its typically aggressive course and rapid progression. Prompt diagnosis and swift progression to definitive treatment, including surgery and adjunctive therapies, become essential components of improving prognostic outcomes. In endometrial cancer, while the direct impact of delays on survival may be less consistently severe, prolonged waiting times remain detrimental, influencing disease progression, patient anxiety, and overall care experience. Clinicians should balance the urgency for treatment initiation with careful patient evaluation and planning to avoid both excessively short and excessively prolonged delays.

Furthermore, the psychological impacts associated with delays underline the necessity for clinicians to prioritize patient communication, actively managing expectations and providing clear timelines and reassurances throughout the diagnostic and treatment journey. Incorporating structured psychological support and counseling services as integral components of cancer care can mitigate distress and improve patient satisfaction. Clinicians must also recognize healthcare system-related barriers that contribute significantly to treatment delays, advocating for timely interventions and resource allocation that address systemic inequities. This includes improving coordination within healthcare services, streamlining referral processes, and enhancing regional access to specialist care. Finally, the COVID-19 pandemic highlighted the importance of developing robust contingency plans and adaptive clinical guidelines to ensure continuity and quality of care during healthcare crises.

In summary, these clinical implications stress the urgent need for systematic improvements in both individual patient management and broader healthcare delivery systems to optimize cancer care timelines, enhance patient outcomes, and ensure equitable access to essential treatments.

### 4.3. Patient-Reported Outcomes and Psychological Impacts

This review highlights significant psychological impacts and alterations in patient-reported outcomes associated with treatment delays. Several studies reported that prolonged waiting periods exacerbated patient anxiety and emotional distress, significantly diminishing quality of life. The uncertainty associated with treatment delays contributed substantially to patient dissatisfaction with healthcare services, underscoring the importance of addressing patient perceptions and experiences throughout the care pathway.

Robinson et al. [23] and Frey et al. [13] emphasized these psychological dimensions, demonstrating that treatment delays not only compromise clinical outcomes but also considerably affect patients’ mental health and emotional well-being. Such findings indicate the critical necessity for healthcare providers to integrate psychological support services, ensure clear and regular communication regarding treatment timelines, and set realistic patient expectations to alleviate anxiety. Moreover, the pandemic-related disruptions investigated by Frey and colleagues further illustrate how external stressors intensify psychological distress. These unprecedented delays magnified patient uncertainty, reinforcing the need for healthcare systems to adopt comprehensive strategies, including psychological counseling and robust communication protocols, to maintain patient trust and satisfaction during times of healthcare disruptions. Therefore, clinicians should be aware that reducing patient distress involves more than shortening wait times; it also requires addressing the psychological aspects comprehensively through empathetic, transparent communication and supportive services tailored to patient needs.

### 4.4. Influence of Healthcare System Factors on Treatment Timeliness

This review identified critical healthcare system-related factors that significantly contributed to treatment delays for ovarian and endometrial cancers. Variations between public and private healthcare sectors, geographic disparities, and socioeconomic inequalities emerged as prominent barriers impacting timely access to cancer treatments. Studies such as those by Frey et al. [12] clearly demonstrated notable differences in treatment intervals between public and private healthcare settings, with public hospitals generally associated with longer delays due to resource constraints and higher patient volumes. Similarly, regional disparities were highlighted by O’Leary et al. [21] and Nica et al. [20], indicating that patients residing in rural or remote areas frequently experienced substantial delays linked to limited specialist availability and logistical challenges.

Moreover, socioeconomic factors, including income level, education, and demographic characteristics, significantly affected patients’ ability to access timely treatment. Studies utilizing large-scale national databases (Shalowitz et al. [25]; Strohl et al. [26]) found pronounced delays disproportionately impacting lower socioeconomic groups and ethnic minorities, underlining systemic inequities within healthcare delivery systems. Addressing these systemic barriers requires targeted interventions, including policy reforms aimed at resource allocation, enhancing accessibility in underserved regions, and implementing effective strategies to overcome socioeconomic disparities. Healthcare systems should focus on developing integrated, coordinated care pathways, ensuring equitable access, and improving overall efficiency and responsiveness to reduce treatment delays and enhance patient outcomes.

### 4.5. Impact of COVID-19 Pandemic on Cancer Care Delivery

The COVID-19 pandemic emerged as a significant factor influencing treatment delays for ovarian and endometrial cancers, highlighting critical vulnerabilities in healthcare systems during global health emergencies. The findings of Frey et al. [13] specifically documented substantial disruptions in scheduled cancer treatments attributable to the pandemic, resulting from the reallocation of healthcare resources, cancellation or postponement of elective surgical procedures, and limitations imposed on outpatient and inpatient care services.

These disruptions caused unprecedented treatment delays, often without predictable timelines, markedly intensifying patient anxiety and negatively affecting quality of life. This unpredictability underscored the essential role of transparent communication and robust contingency planning to minimize psychological distress and maintain continuity of care during crises.

### 4.6. Methodological Variability and Study Heterogeneity

One of the notable challenges encountered in interpreting the results of this systematic review was the considerable methodological variability and heterogeneity among included studies. This variability manifested primarily in differing definitions and measurements of treatment delays, ranging widely from initial symptom recognition to definitive diagnosis and subsequent treatment initiation. For instance, delays were reported variably as median or mean days, specific cutoff intervals (such as six, eight, or twelve weeks), or general categorical descriptors, complicating direct comparison and synthesis across studies.

Moreover, studies differed significantly in their choice of outcomes assessed, including survival metrics, cancer staging, and patient-reported measures. Some studies prioritized clinical outcomes such as survival rates and disease progression, while others focused more on patient-centric outcomes such as psychological distress, quality of life, and satisfaction. Such diverse outcome reporting limited the ability to conduct quantitative meta-analyses and required reliance primarily on qualitative narrative synthesis.

Differences in patient populations further contributed to heterogeneity, with studies spanning varied geographic regions, healthcare systems, and demographic backgrounds. For example, findings from high-resource settings often differed substantially from those in lower-resource settings, potentially due to differences in healthcare infrastructure, access to specialized services, and patient socioeconomic factors.

Additionally, study designs across included research ranged from hospital-based retrospective cohort analyses to large-scale, population-based studies using national registries. While large registry-based studies offered extensive datasets and robust statistical power, smaller hospital-based studies provided detailed clinical insights and nuanced perspectives on patient experiences. Each study design presented its own set of methodological strengths and limitations, influencing the overall risk of bias and applicability of findings.

This considerable methodological heterogeneity underscores the necessity of standardizing definitions, measures, and reporting practices for future research. Establishing consistent guidelines for measuring and reporting delays, clearly defining critical intervals, and adopting uniform outcome criteria would significantly enhance comparability across studies. Additionally, prioritizing comprehensive reporting of both clinical and patient-centered outcomes could provide a balanced perspective, further enriching the interpretation and clinical utility of research findings. Addressing these methodological discrepancies is vital for accurately assessing the true impacts of treatment delays and effectively guiding clinical practice and healthcare policy.

### 4.7. Risk of Bias and Strength of Evidence

This review systematically evaluated the risk of bias across included studies using the ROBINS-E tool, highlighting variations in the methodological rigor of the examined research. Most studies included in the analysis were categorized as having a moderate risk of bias, indicating that the findings are generally credible but should be interpreted cautiously due to potential confounding factors and limitations related to participant selection or exposure measurement. Several studies, notably those conducted by Crawford et al. [10], Hansen et al. [27], and Rabiu et al. [22], demonstrated a serious overall risk of bias. Key concerns contributing to this classification included significant issues with confounding variables, participant selection biases, missing data, and potential inaccuracies in outcome or exposure measurement. Such methodological limitations considerably weaken confidence in their findings and underline the necessity for careful consideration when incorporating these studies into clinical decision-making or policy formulation.

In contrast, studies derived from large national databases and registries, such as those conducted by AlHilli et al. [9], Shalowitz et al. [25], and Strohl et al. [26], generally exhibited lower risk of bias, primarily due to robust participant selection processes, comprehensive data collection, and rigorous outcome reporting standards. Nevertheless, even these studies are susceptible to residual confounding due to their observational nature, warranting cautious interpretation.

Overall, the strength of evidence presented in this review varies considerably depending on the specific outcomes assessed and the methodological quality of individual studies. Given the inherent limitations of observational research, particularly concerning confounding and selection biases, findings should be considered indicative rather than definitive. Future studies with rigorous methodological approaches, including prospective designs and standardized protocols, are necessary to strengthen the evidence base and provide more reliable guidance for clinical practice and healthcare policy.

### 4.8. Strengths and Limitations of the Review

This systematic review possesses several strengths, notably its comprehensive literature search across multiple databases, rigorous selection criteria, and detailed evaluation of study quality using the ROBINS-E tool. Additionally, the inclusion of studies from diverse geographical regions enhances the generalizability of findings. Nevertheless, the review has limitations, primarily due to significant methodological heterogeneity among studies, which precluded quantitative meta-analysis and necessitated reliance on qualitative narrative synthesis. Furthermore, the potential for publication and reporting biases, particularly given the reliance on observational studies, should also be acknowledged as a limitation impacting the certainty of conclusions drawn.

### 4.9. Recommendations for Clinical Practice and Policy

Based on the findings of this systematic review, several key recommendations can be proposed for clinical practice and healthcare policy. Clinicians should prioritize minimizing delays from diagnosis to treatment, particularly within the critical six- to eight-week window, to optimize patient outcomes. Clear, consistent, and empathetic communication strategies should be integrated into clinical practice to reduce patient anxiety and enhance satisfaction. Healthcare policymakers should address systemic inequities by improving resource allocation, enhancing regional healthcare infrastructure, and ensuring equitable access to specialist care. Contingency planning and the establishment of robust, flexible guidelines are recommended to mitigate the impact of future healthcare disruptions similar to the COVID-19 pandemic.

## 5. Conclusions

This systematic review provides evidence demonstrating that treatment delays significantly impact clinical outcomes in ovarian and endometrial cancer, notably influencing survival rates, disease progression, and patient psychological well-being. The findings underline the importance of timely interventions, particularly within critical intervals such as the identified six- to eight-week window following diagnosis. Differences between ovarian and endometrial cancers were evident, highlighting a more pronounced urgency in ovarian cancer due to its aggressive nature.

Patient experiences are markedly affected by delays, with heightened psychological distress, anxiety, and reduced overall satisfaction with healthcare services reported frequently. Healthcare system factors, including resource allocation disparities, geographical inequalities, and socioeconomic factors, were identified as important determinants of timely cancer care. The unprecedented disruptions caused by the COVID-19 pandemic further exposed vulnerabilities in cancer care delivery, highlighting the critical need for meticulous planning and crisis management protocols to maintain continuity and quality of care.

The methodological inconsistencies and variability among studies reviewed represent a notable challenge that emphasizes the need for the standardization of delay measurements, clear reporting guidelines, and consistent outcome definitions in future research. Addressing these methodological issues would significantly strengthen the quality and reliability of evidence, enhancing its applicability in clinical and policy decision-making.

In conclusion, reducing delays in the treatment of ovarian and endometrial cancers requires systematic improvements in clinical practices, patient communication, psychological support, and healthcare infrastructure. Continued efforts should focus on addressing research gaps, refining methodological approaches and promoting policies aimed at decreasing delays to ultimately improve clinical outcomes and equity in cancer care.

## Figures and Tables

**Table 1:** Endometrial cancer studies.

Study Author	Year	Country	Study Design	Sample Size	Study Setting	Delay Measurement	Outcomes Evaluated
AlHilli et al. [9]	2019	USA	Retrospective cohort	284,499	Population-based	Diagnosis to surgery	Survival outcomes, stage progression
Crawford et al. [10]	2002	UK	Retrospective cohort	703	Hospital-based	Referral/diagnosis to treatment	Survival outcomes
Elit et al. [11]	2014	Canada	Population-based cohort	9417	Population-based	Histologic diagnosis to hysterectomy	Survival outcomes, stage progression
Frey et al. [12]	2016	Australia	Retrospective cohort	143 (Endometrial subgroup)	Hospital-based	Diagnosis to surgery	Delay intervals (public vs. private hospitals)
Kadan et al. [15]	2020	Israel	Retrospective cohort	468	Hospital-based	Biopsy diagnosis to surgery	Adjuvant therapy needs, survival outcomes
Levin et al. [16]	2024	Canada	Retrospective cohort	160	Hospital-based	Biopsy diagnosis (atypical hyperplasia) to surgery	Presence of concurrent carcinoma
Marcickiewicz et al. [17]	2022	Sweden	Retrospective cohort	7366	Population-based	Diagnosis to primary surgery	Survival outcomes, sociodemographic predictors of delay
Matsuo et al. [18]	2015	USA	Retrospective cohort	435	Hospital-based	Biopsy diagnosis to surgical staging	Survival outcomes, tumor grade upgrading
Mitric et al. [19]	2020	Canada	Retrospective cohort	136	Hospital-based	Biopsy diagnosis to surgery	Survival outcomes, tumor aggressiveness
Nica et al. [20]	2022	Canada	Retrospective cohort	3518	Population-based	Diagnosis and oncology consultation to surgery	Survival outcomes
O’Leary et al. [21]	2013	Canada	Population-based cohort	9330	Population-based	Diagnosis to surgery	Delay intervals trends, predictors of delay
Robinson et al. [23]	2012	Denmark	Nationwide cohort	165 (Endometrial subgroup)	Population-based	Symptoms to diagnosis/treatment	Quality of life, patient satisfaction, survival
Sabourin et al. [24]	2015	Canada	Retrospective cohort	2809	Population-based	Histologic diagnosis to surgery	Survival outcomes, stage progression
Shalowitz et al. [25]	2017	USA	Retrospective cohort	208,438	Population-based	Diagnosis to surgery	Survival outcomes, optimal surgical timing
Strohl et al. [26]	2016	USA	Retrospective cohort	112,041	Population-based	Diagnosis to definitive surgery	Survival outcomes, demographic disparities
Zouzoulas et al. [28]	2024	Greece	Retrospective cohort	259	Hospital-based	Biopsy diagnosis to surgery	Survival outcomes

**Table 2:** Ovarian cancer studies.

Study Author	Year	Country	Study Design	Sample Size	Study Setting	Delay Measurement	Outcomes Evaluated
Frey et al. [12]	2016	Australia	Retrospective cohort	114 (Ovarian subgroup)	Hospital-based	Diagnosis to surgery	Delay intervals (public vs. private hospitals)
Frey et al. [13]	2020	Australia	Retrospective cohort	555	Hospital-based	COVID-19 related delay in planned treatment	Patient anxiety, QoL impacts
Hansen et al. [27]	2011	Denmark	Population-based cohort	59	Population-based	Symptoms to definitive treatment	Delay intervals, survival outcomes
Huepenbecker et al. [14]	2022	Germany	Retrospective cohort	13,872	Population-based	Symptoms to diagnosis and diagnosis to treatment	Survival outcomes
Rabiu et al. [22]	2013	Nigeria	Retrospective cohort	37	Hospital-based	Symptoms to definitive treatment	Stage at diagnosis, treatment compliance
Robinson et al. [23]	2012	Denmark	Nationwide cohort	188 (Ovarian subgroup)	Population-based	Symptoms to diagnosis/treatment	Quality of life, patient satisfaction, survival
Zouzoulas et al. [29]	2025	Greece	Retrospective cohort	72	Hospital-based	Symptoms to definitive treatment	Survival outcomes

**Table 3:** Data summary table of included studies.

Study Author	Year	Cancer Type	Sample Size	Delay Interval	Delay Measurement	Survival Outcomes	Disease Progression/Stage Outcomes	Patient-Reported Outcomes	Healthcare System Factors
AlHilli et al. [9]	2019	Endometrial	284,499	Dx–Tx	>6 weeks associated with poorer survival	↓ survival	Stage progression linked to delays	-	-
Crawford et al. [10]	2002	Endometrial	703	Referral–Tx	Median delay: 43 days	↓ survival with longer delays	Advanced stage at longer delays	-	Potential systemic delays
Elit et al. [11]	2014	Endometrial	9417	Dx–Tx	Median delay: 36 days	↓ survival with increased delay	Advanced stage correlated with increased delay	-	Population-based delays
Frey et al. [12]	2016	Endometrial, ovarian	257	Dx–Tx	Median delay (endo): 34 days; (ov): 28 days	-	-	Reduced satisfaction in public hospitals	Longer delays in public hospitals
Frey et al. [13]	2020	Ovarian	555	COVID-related	Significant delays due to COVID-19	-	-	↑ anxiety, ↓ QoL	Healthcare disruptions due to pandemic
Hansen et al. [27]	2011	Ovarian	59	Sx–Tx	Median delay: 100 days	↓ survival, delays detrimental	Advanced stage at diagnosis due to delays	-	Systemic and patient delays
Huepenbecker et al. [14]	2022	Ovarian	13,872	Sx–Dx–Tx	Sx–Dx (median 66 days) Dx–Tx (median 29 days)	↓ survival linked to symptom delays	Advanced disease linked to symptom delays	-	German healthcare registry data
Kadan et al. [15]	2020	Endometrial	468	Dx–Tx	Median delay: 30 days	Minimal survival impact	Minimal impact on stage progression	-	-
Levin et al. [16]	2024	Endometrial	160	Dx–Tx	Median delay: 42 days	-	Minimal stage progression	-	-
Marcickiewicz et al. [17]	2022	Endometrial	7366	Dx–Tx	Optimal interval: 2–6 weeks	↓ survival at extremes	-	-	Socioeconomic disparities affecting delays
Matsuo et al. [18]	2015	Endometrial	435	Dx–Tx	Median delay: 34 days	No clear survival disadvantage	Minimal grade/stage progression	-	-
Mitric et al. [19]	2020	Endometrial	136	Dx–Tx	Median delay: 42 days	No significant survival disadvantage	Minimal disease aggressiveness impact	-	-
Nica et al. [20]	2022	Endometrial	3518	Dx–Tx	Median delay: 45 days	↓ survival with increased delay	-	-	Regional disparities, healthcare system impacts
O’Leary et al. [21]	2013	Endometrial	9,33	Dx–Tx	Increased delay trends	↓ survival with longer delays	Increased advanced-stage cases with delays	-	Regional variations, resource availability
Rabiu et al. [22]	2013	Ovarian	37	Sx–Tx	Severe delays (>3 months)	-	Advanced stage at presentation	-	Socioeconomic factors affecting delay
Robinson et al. [23]	2012	Endometrial, ovarian	453	Sx–Tx	Median delay (endo): 77 days; (ov): 92 days	Poorer survival in ovarian cancer	Advanced stage due to delays	↓ QoL, patient dissatisfaction	System delays, socioeconomic factors
Sabourin et al. [24]	2015	Endometrial	2809	Dx–Tx	Median delay: 43 days	↓ survival with delays	Increased stage progression	-	Canadian provincial healthcare
Shalowitz et al. [25]	2017	Endometrial	208,438	Dx–Tx	Optimal interval: 3–8 weeks	↓ survival outside optimal interval	-	-	Socioeconomic disparities, demographic impacts
Strohl et al. [26]	2016	Endometrial	112,041	Dx–Tx	Delays associated with demographic disparities	↓ survival with longer delays	-	-	Disparities based on socioeconomic/racial factors
Zouzoulas et al. [28]	2024	Endometrial	259	Dx–Surgery	>8 weeks	Worse DFS, no difference in OS	Increased need for adjuvant pelvic radiation	-	Older age, higher BMI, and more comorbidities in delayed group
Zouzoulas et al. [29]	2025	Ovarian (early stage)	72	Dx–Surgery	>5 weeks	No significant differences in DFS or OS	No significant differences in stage progression	-	No significant differences in postoperative complications

Dx: diagnosis, Tx: treatment, Sx: symptoms, ↓: decrease and ↑: increase.

**Table 4:** Summary table of quantitative data.

Study Author	Year	Cancer Type	Delay Interval	Survival Outcomes/Quantitative Data
AlHilli et al. [9]	2019	Endometrial	Dx–Tx (>6 weeks)	Stage I-II: HR 1.22 (95% CI 1.16–1.29) Stage III: HR 0.99 (95% CI 0.91–1.08, non-significant) Stage IV: improved survival (HR 0.89; 95% CI 0.80–0.99)
Shalowitz et al. [25]	2017	Endometrial	Dx–Tx (>8 weeks or <2 weeks)	Surgery within 1 week HR 1.4 (95% CI 1.3–1.5); within 2 weeks HR 1.1 (95% CI 1.0–1.2). Increased mortality risk.
Strohl et al. [26]	2016	Endometrial	Dx–Tx (>6 weeks)	Overall survival decrease: HR 1.14 (95% CI 1.09–1.20)
Crawford et al. [10]	2002	Endometrial	Referral–Tx (>40 days)	62–91 days HR 0.47 (95% CI 0.27–0.83); >92 days HR 0.53 (95% CI 0.30–0.93)
Elit et al. [11]	2014	Endometrial	Dx–Surgery (>12 weeks or ≤2 weeks)	Significantly worse 5-year OS for delays >12 weeks; for delays ≤2 weeks, 5-year OS 71.1% (also poorer outcome)
Matsuo et al. [18]	2015	Endometrial	Dx–Tx intervals (1–14, 15–42, 43–84, and ≥85 days)	No significant differences in survival (5-year OS rates 62.5%, 93.6%, 95.2%, and 100%, respectively)
Mitric et al. [19]	2020	Endometrial	Dx–Tx (>12 weeks)	No significant impact on DFS (HR 1.2; 95% CI 0.6–2.5), OS (HR 1.1; 95% CI 0.6–2.1), or PFS (HR 0.9; 95% CI 0.5–1.7)
Nica et al. [20]	2022	Endometrial	First oncology appointment to Surgery (>45 days)	46–60 days HR 1.19 (95% CI 1.04–1.36); 61–75 days HR 1.42 (95% CI 1.11–1.83)
Sabourin et al. [24]	2015	Endometrial	Dx–Surgery (>12 weeks)	5-year OS: ≤6 weeks 87.1%, 6–12 weeks 84.1%, >12 weeks 79.8%; delay >12 weeks HR 1.41 (95% CI 1.03–1.93)
Zouzoulas et al. [28]	2024	Endometrial	Dx–Surgery (>8 weeks)	DFS significantly worse with delays >8 weeks (*p* = 0.0312); OS not significantly impacted (*p* = 0.146)
Zouzoulas et al. [29]	2025	Ovarian (early stage)	Dx–Surgery (>5 weeks)	No significant impact on DFS (*p* = 0.48) or OS (*p* = 0.703)

Dx: diagnosis, Tx: treatment, and Sx: symptoms.

## Data Availability

In accordance with the journal’s guidelines, the data presented in this study are available on request from the corresponding author for the reproducibility of this study if such is requested.

## Abbreviations

The following abbreviations are used in this manuscript:

**BMI**	**Body Mass Index**
QoL	Quality of Life
COVID-19	Coronavirus Disease 2019
ROBINS-E	Risk Of Bias In Non-randomized Studies of Exposures
HR	Hazard Ratio
OR	Odds Ratio
CIs	Confidence Intervals
GRADE	Grading of Recommendations Assessment, Development, and Evaluation
Dx	Diagnosis
Tx	Treatment
Sx	Symptoms
OS	Overall Survival
DFS	Disease-Free Survival
